# The R403Q Myosin Mutation Implicated in Familial Hypertrophic Cardiomyopathy Causes Disorder at the Actomyosin Interface

**DOI:** 10.1371/journal.pone.0001123

**Published:** 2007-11-07

**Authors:** Niels Volkmann, HongJun Lui, Larnele Hazelwood, Kathleen M. Trybus, Susan Lowey, Dorit Hanein

**Affiliations:** 1 Burnham Institute for Medical Research, La Jolla, California, United States of America; 2 Department of Molecular Physiology and Biophysics, University of Vermont, Burlington, Vermont, United States of America; Children's Hospital Boston, United States of America

## Abstract

**Background:**

Mutations in virtually all of the proteins comprising the cardiac muscle sarcomere have been implicated in causing Familial Hypertrophic Cardiomyopathy (FHC). Mutations in the β-myosin heavy chain (MHC) remain among the most common causes of FHC, with the widely studied R403Q mutation resulting in an especially severe clinical prognosis. *In vitro* functional studies of cardiac myosin containing the R403Q mutation have revealed significant changes in enzymatic and mechanical properties compared to wild-type myosin. It has been proposed that these molecular changes must trigger events that ultimately lead to the clinical phenotype.

**Principal Findings:**

Here we examine the structural consequences of the R403Q mutation in a recombinant smooth muscle myosin subfragment (S1), whose kinetic features have much in common with slow β-MHC. We obtained three-dimensional reconstructions of wild-type and R403Q smooth muscle S1 bound to actin filaments in the presence (ADP) and absence (apo) of nucleotide by electron cryomicroscopy and image analysis. We observed that the mutant S1 was attached to actin at highly variable angles compared to wild-type reconstructions, suggesting a severe disruption of the actin-myosin interaction at the interface.

**Significance:**

These results provide structural evidence that disarray at the molecular level may be linked to the histopathological myocyte disarray characteristic of the diseased state.

## Introduction

Heart failure is a world wide public health problem that affects several million patients in the United States alone [reviewed in 1]. One prime cause of heart disease is familial hypertrophic cardiomyopathy (FHC), which is an inherited cardiac disease that frequently results in sudden death of young and otherwise healthy individuals [reviewed in 2]. The clinical manifestation of FHC is widely varied. Common features include asymmetric septal hypertrophy, potential outflow tract obstruction, foci of disorganized myocytes, cellular disarray of the contractile apparatus, myofibril disarray, interstitial fibrosis and arrhythmia [3]. Even in the presence of markedly abnormal ventricular morphology and histopathology, contractile (systolic) function of the patients' heart is usually excellent, and can often appear supra normal. Although the anatomic, histologic and cellular findings in FHC are well described, the molecular events that trigger compensatory forms of cardiac remodeling remain largely unknown.

The majority of FHC cases have been linked to single point mutations in various contractile proteins of the cardiac sarcomere. Most of the known (>100) β-myosin heavy chain (MHC) mutations have been located in the globular head of myosin (subfragment 1, S1) or near the head-rod junction [4], [5]. Many of these residues are conserved in a wide variety of myosin II isoforms, suggesting that they may be important for normal function of the molecule.

The myosin point mutation of Arg^403^ to glutamine (R403Q) causes one of the most severe phenotypes of FHC. Fifty percent of the affected individuals die by 40 years of age. Arg^403^ is located at the base of a surface loop (the “cardiomyopathy loop”) that was shown to directly interact with actin (near its nucleotide binding cleft) for several myosin isoforms including skeletal and smooth muscle myosin II [6], [7], [8]. The charge of the residue is conserved (Arg or Lys) for most published myosin sequences (>40). Thus, R403Q is one of the few FHC mutations that can directly affect actin binding. The effect of FHC mutations (and of R403Q in particular) on myosin activity has been studied predominantly either by *in vitro* analysis of myosin motor activity [9], [10], [11], [12], [13], [14], [15] or by animal models of the disease [16], [17], [18]. A consistent finding from the analyses indicates that the R403Q cardiac phenotype is due to the dominant effects of the mutant on sarcomere function. It remains unclear how altered functional activity can lead to the morphological disarray observed for FHC hearts. Is this malignant myosin mutation directly disrupting myofibril assembly through molecular level changes, or is this disarray a secondary effect of changes in global cardiac function and loading?

The primary aim of the present study is to investigate the structural consequences of introducing a missense mutation into the “myopathy loop” (Arg^403^) of a vertebrate smooth muscle myosin. A smooth muscle myosin fragment was expressed in the baculovirus/insect cell system [15]. Although human β-cardiac myosin would be clearly the isoform of choice for our studies, expression levels of human β-cardiac myosin heavy chain [19] and of rat α-cardiac myosin [13] have been very low, and thus are not amenable to extensive biochemical and structural studies. The main source of mutated cardiac myosin is from transgenic mice, but they express primarily α-cardiac myosin in the ventricles throughout adult life. The functional consequences of mutations in a fast myosin isoform, such as mouse α-cardiac myosin, may well be different from those in β-cardiac myosin, a slow myosin isoform. Despite sequence differences, smooth muscle myosin is in many respects functionally similar to β-cardiac myosin, insofar as they both share low values of actin-activated ATPase activity, actin translocation velocity, and kinetic constants [20], [21], [22].

An important advantage in studying point mutations in the smooth muscle heavy chain is that the crystal structure of the expressed motor domain with its associated essential light chain was solved by X-ray crystallography to atomic resolution [23]. In addition, atomic models of the smooth muscle myosin-actin complex in two nucleotide states (apo and ADP) were derived by fitting atomic structures of actin and of smooth muscle myosin domains into 3D reconstructions obtained by electron cryomicroscopy [8]. The same study showed that there is a significant change in the actin interactions of the cardiomyopathy loop if the apo and ADP structures are compared. This change was not observed for the faster skeletal myosin isoforms [6] and may be a feature common to slow myosins.

The atomic structures of smooth muscle myosin domains are very similar to those of the chicken skeletal isoform [24]. The structures of more remotely related myosins such as myosin V [25], [26]and myosin I [27] are also very similar, indicating that the major domain structures are conserved among all myosins. In addition, the sequence of the cardiomyopathy loop is well conserved between β-cardiac and smooth muscle myosins, making smooth muscle myosin an appropriate model system for characterizing changes caused by mutations of Arg^403^.

We generated 3D reconstructions of wild-type and R403Q smooth muscle myosin S1 (R406Q in the smooth muscle sequence) bound to actin in the apo and ADP states using electron cryomicroscopy and image analysis. Consistent with its close proximity to elements involved in forming the actin interface [8], we observe that the R403Q mutation severely disrupts actin-myosin interactions when compared to the wild-type reconstructions. The usually fixed attachment angle of myosin to the actin filament is much less well defined with a random deviation of about 15° in the presence of the mutation for both apo and ADP states. The nucleotide dependent internal conformation of myosin while attached to actin does not appear to be affected. Our results indicate that the histopathology hallmark of FHC, namely the sarcomeric disarray, may be directly linked to molecular level variability of the R403Q myosin molecule while complexed with actin.

## Results

The R403Q mutation is the most widely studied FHC-related mutation that is located in close proximity to the actin-binding interface of myosin. We generated reconstructions of smooth muscle myosin S1 carrying the R403Q mutation attached to actin filaments using electron cryomicroscopy and image analysis. We obtained reconstructions in the absence of nucleotide (apo state) and in the presence of MgADP (ADP state). We compared these 3D maps to reconstructions of wild-type smooth muscle actomyosin obtained in parallel, and to previously obtained wild-type maps [8].

### Reconstructions of R403Q myosin bound to actin filaments in the apo and ADP states differ significantly

**Figure 1:**
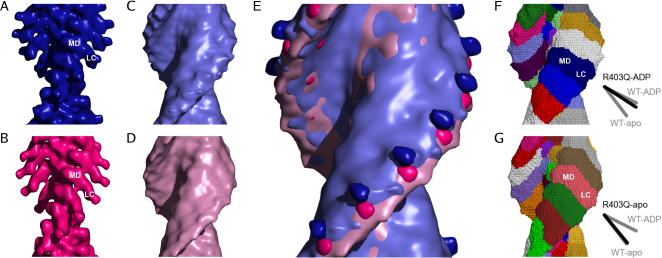
Surface representations of smooth muscle actomyosin constructs. The pointed end of the filaments is towards the top of the figure. A: Wild-type smooth muscle actomyosin in the presence of ADP. B: Wild-type smooth muscle actomyosin in the apo state. The contour level for A and B is chosen to represent the correct molecular mass. Note the well defined density and angle of the light-chain region (LC) C: R403Q mutant smooth muscle actomyosin in the presence of ADP. D: R403Q mutant smooth muscle actomyosin in the apo state. The contour level for C and D is chosen to show as much of the light chain domains as possible without completely obscuring the shape of the motor domain. E: Overlay of the maps in A–D. Color code and contouring as in A–D. F, G: Watershed segmentation of the maps in C (F) and D (G). These results reconfirm the orientation of the light-chain domains that correspond to those of the wild-type reconstructions (sketches) and the better definition of boundaries in the center of the filaments: the actin subunits are well segmented while there is no sub-segmentation of myosin domains as can be obtained for wild-type reconstructions [33]. The sketches show central lines extracted from the density of the wild-type (grey) and the segmentation of the R403Q (black). Only the line segments extracted for the corresponding light-chain regions (LC) are shown, line segments corresponding to the motor domain region (MD) overlap almost completely for all maps.

**Figure 2:**
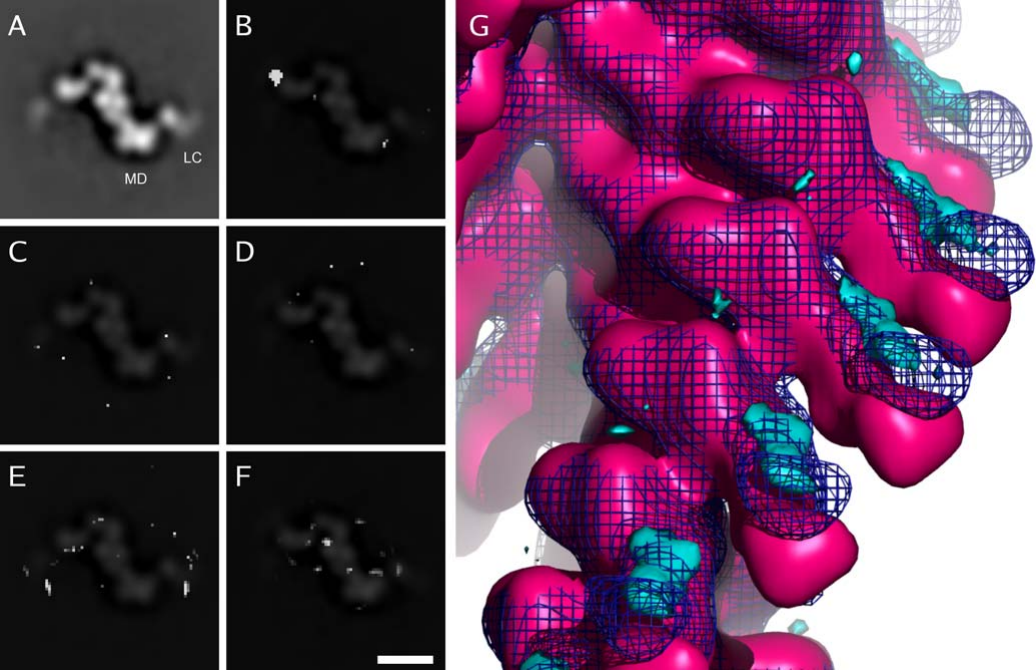
Difference and structural variability maps. 4-nm wide slices perpendicular to the helix axis of several maps are shown on the left. Only peaks significant at a confidence level of 99.99% are shown. A: Wild-type smooth muscle actomyosin in the absence of nucleotide. The motor domain (MD) and light-chain (LC) regions are labeled. A faint ghost image of this map is also overlaid on C and E to aid visualization. B: A difference map generated by subtracting the R403Q mutant smooth muscle actomyosin apo state reconstruction from the R403Q mutant smooth muscle actomyosin ADP state reconstruction. A clear difference peak can be identified in the light-chain region. A faint ghost image of the wild-type ADP state reconstruction is overlaid to aid visualization. This image is also overlaid on D and F. C: Difference map between two independently generated R403Q mutant apo state reconstructions. Only occasional, randomly distributed, isolated pixels can be seen, no coherent difference peaks exist. D: Difference map between two independently generated R403Q mutant ADP state reconstructions. E: Structural variability (AVID map) of R403Q mutant apo state reconstruction. Only randomly distributed peaks can be seen, there is no consistent structural variability in any confined region. F: Structural variability (AVID map) of R403Q mutant ADP state reconstruction. Bar:10 nm. G: Surface representation of the difference map shown in B. The cyan density represents additional density in the R403Q mutant ADP state reconstruction if compared to the R403Q mutant apo state reconstruction. The apo state (pink) and ADP state (blue wireframe) wild-type reconstructions are also shown. The difference between the mutant reconstructions is located in the light-chain region and correlates with the changes observed in wild-type smooth muscle actomyosin.

We generated 3D maps of wild-type actomyosin complexes as well as actomyosin containing R403Q smooth muscle S1 in the apo state and in the ADP state under cryo-conditions (Figure 1 and Table 1). Each data set was divided into two separate clusters based on the similarity of their layer-line patterns. The near and far sides of the Fourier spectrum were kept separate for each of these reconstructions. Thus, for each condition four independent helical reconstructions were calculated. The subdivided data sets were also processed by using the modified version [7] of the iterative helical real space refinement (IHRSR) method [28] resulting in another two reconstructions per condition. This process was repeated using an independent biochemical preparation. Using the Student's t-test, we found that there was no statistically significant difference (P<0.005) between the twelve R403Q reconstructions within each condition (Figure 2C,D), but a statistically significant difference between the apo and the ADP state (Figure 2B). Interestingly, this difference is closely correlated with the difference between the wild-type reconstructions in the ADP and apo states (Figure 2G), that shows a downward movement of the light-chain region when no nucleotide is bound. This movement is not directly apparent for the R403Q maps, but the difference maps indicate that a similar movement has taken place.

**Table 1:** Data collection

No. of Data Sets^a^	WT, rigor	WT, ADP	R403Q, rigor	R403Q, ADP
	6	6	12	12
**Helical Reconstructions**				
Subunits in Averages	5028±1754^b^	5866±1887	9245±2903	6225±2267
Subunits per Turn	2.160±0.003	2.160±0.004	2.158±0.003	2.160±0.003
Phase Residual (°)^c^	25±5	24±6	45±8	38±7
**Iterative Helical Real Space Refinement**				
Subunits in Averages	11012±3113	8422±2540	16952±2685	13848±3650
Subunits per Turn	2.161±0.005	2.159±0.006	2.161±0.003	2.163±0.004

a This counts split data (i.e. far and near side) as different data sets (see text for details).

b All statistics are calculated over the respective number of data sets.

c The phase residual is a measure for the homogeneity and quality of the data. Values below 45° are considered excellent; values below 55° are acceptable.

### Reconstructions of R403Q myosin bound to actin filaments have randomized attachment angles in both states

**Figure 3:**
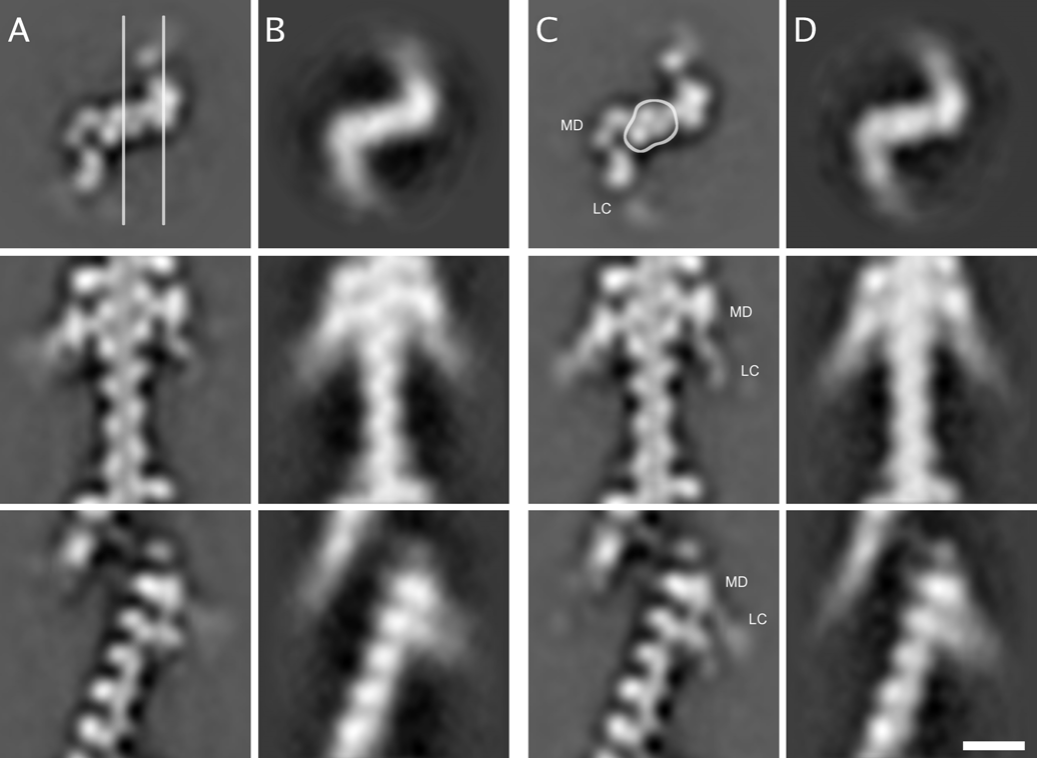
Comparison between actin decorated with wild-type smooth muscle myosin S1 and actin decorated with a R403Q smooth muscle myosin mutant. A: Wild-type smooth muscle actomyosin in the presence of ADP. The top row shows a 4-nm thick central slice perpendicular to the filament axis. The second row shows a 4-nm thick central slice parallel to the filament axis (cutting plane indicated by the left line in top row). The pointed end is to the top of the figure in the two lower rows. The bottom row shows an adjacent 4-nm thick slice (cutting plane indicated by right line in top row). This slice goes primarily through the S1 molecules. B: R403Q mutant smooth muscle actomyosin in the presence of ADP. Organization of rows as in (A). C: Wild-type smooth muscle actomyosin in the absence of nucleotide (apo). The approximate location of the motor domain (MD) and the light chain (LC) regions are labeled in each view. Organization of rows as in A. The approximate outline of the actin portion of the filament is indicated. D: Apo R403Q mutant smooth muscle actomyosin. Organization of rows as in (A). Bar:10 nm.

We checked the density distributions of each mutant reconstruction for signs of partial decoration or mixed populations, which would change the relative strength of features in the maps. Regions of the underlying structure that are not well locked into space spread their density over a larger area and would appear weaker than well-determined entities. In the reconstructions done for the actin filaments decorated with wild-type smooth muscle, the relative strengths of actin, the motor domain, and the light chains are comparable for both the ADP and apo states (Figure 3A,C). In contrast, the reconstructions from the R403Q mutant display a more widely spread density within the whole myosin area for both states (Figure 3B,D).

To ensure that this effect is not caused by partial decoration of the filaments (as opposed to a true mix of conformations), we carefully analyzed the appearance of the filaments before we selected them for averaging. Partial decoration would be immediately apparent in the images by disruptions of the characteristic arrow-head appearance of myosin-decorated actin filaments. We selected only filaments that clearly showed full decoration. Again, these new reconstructions showed the same relative change in the strength of the features in the maps, reinforcing the notion that this effect is not due to partial decoration.

To further test for partial decoration in the resulting reconstructions, we determined the Absolute Values of Individual Differences (AVID) for the helical reconstructions. Because AVID measures the variance between asymmetric units, partial decoration leads to an AVID map where there is no density in the area of the filament being decorated but clear density in the area where partial occupancy occurs. The AVID density in that area would be strongest in the case of 50% occupancy because that maximizes the variance, but higher (or lower) occupancies are also clearly detectable (up to ∼90%). The AVID density would encase the whole region that experiences partial occupancy, leading to a “ghost image” of the entire decorating molecule [29]. For neither the apo nor the ADP states of the R403Q reconstructions, did the AVID maps show any indication for ghosting of the myosin density (Figure 2E,F). Thus, the differences between 3D reconstructions for actin filaments decorated with wild-type smooth muscle S1 and those containing the R403Q mutation are solely due to changes in variability in the entire myosin region and not due to partial decoration.

The fact that we could not separate distinct subpopulations using sorting strategies either based on the similarity of the optical diffraction patterns or based on different lever-arm angles indicates that the actin filaments with R403Q myosin do not contain short order (as for example the two states of myosin V in the presence of AMP.PNP; [7] but express truly uncorrelated random mixtures of attachment angles. Modeling of this angular disorder indicates a randomization of the angle with a standard deviation of ∼15°.

### The R403Q mutation does not appear to disturb the nucleotide dependent conformation of the light- chain domain while attached to actin filaments

All R403Q maps (apo and ADP) show an orientation of the light-chain domain that resembles the post-powerstroke position, similar to those obtained for actin bound myosin II [6], [8], [30], [31], [32]. While the mixture of the attachment angles obscures the orientation in the maps, a segmentation analysis using the watershed transform [33] clearly demonstrates that the orientation of the light-chain domains is nucleotide dependent and matches those of the wild-type reconstructions (Figure 1F,G). The watershed transform divides density into natural units and defines the boundary between them, even if they are touching. The application of this method to our data shows (i) that the quality of the map is sufficient to segment actin subunits in the filament core and (ii) that the boundary between the attached myosin molecules displays the same angle as the lever arm visible in the wild-type myosin molecules: the angle of the ADP-state boundaries points downwards to a lesser extent than that of the apo state (Figure 1F,G, sketches). This is also supported by the difference map between the R403Q reconstructions in the ADP and apo states that is highly correlated with the change of the light-chain domain in the wild-type maps (Figure 2G).

## Discussion

**Figure 4:**
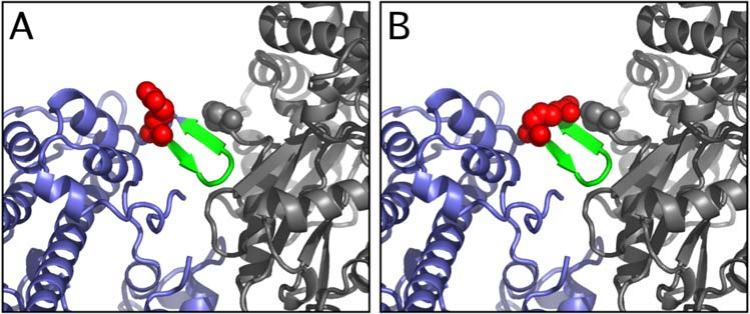
Proximity of residue Arg^406^ (Arg^403^ in cardiac myosin) to the actin interface. Arg^406^ (red spheres) is immediately adjacent to residues of the cardiomyopathy loop that were previously implicated in actin binding by docking studies (407–414; green). While the conformation of the Arg^406^ in the smooth muscle myosin crystal structure points away from the interface (A), it can easily reach actin by simple, stereochemically permitted bond angle rotations (B). The resulting conformation does not generate serious clashes with other myosin residues. Myosin is shown in blue, the interacting actin filament subunits in grey. Residue Pro^333^ of actin, the closest to myosin Arg^406^, is shown as spheres.

Familial hypertrophic cardiomyopathy (FHC) is frequently associated with mutations in the β-cardiac myosin heavy chain. Many of the implicated residues are located in highly conserved regions of the myosin II class, suggesting that these mutations may impair the basic functions of the molecular motor. Only one of these mutations, the R403Q point mutation, located in a region of six conserved amino acids (402–407; PRVKVG), is close enough to the actin surface to potentially participate in actomyosin interactions (Figure 4). The similarity of the attachment angle of myosin II in actomyosin reconstructions [6], [8], [30], [31], [32] with those of other isoforms including myosin V [7], myosin VI [34], and brush border myosin I [35] indicates that this interaction is isoform independent. Thus, this lethal single point mutation might impair myosin-actin basic functions through structural changes in the actomyosin complex. To test this hypothesis, we have prepared recombinant smooth muscle S1 with the R403Q mutation using a baculovirus/insect cell expression system, and have generated 3D reconstructions of R403Q–actomyosin complexes in the two strong binding states (apo and ADP). Compared to the 3D maps that were obtained for wild-type smooth muscle actomyosin complexes, the R403Q maps show a high degree of conformational variability.

*In vitro* characterization of expressed smooth muscle myosin that contains the R403Q missense mutation showed an increase in actin filament velocity in a motility assay and an enhanced actin-activated ATPase activity [15]. Single molecule mechanics using a laser trap, gave a unitary displacement and force for the mutant that was similar to wild-type, but the attachment time to actin following the unitary displacement was markedly reduced. The structural data presented here, indicate that the main factor decreasing the attachment time of the myosin motor domain is a general increase in variability in the actomyosin system. In contrast to wild-type constructs, the reconstructions do not exhibit a well defined attachment angle, but a mixture of a large number of angles. While the wild-type orientation of the light-chain domain appears to be maintained, this large decrease in stability provides a structural basis for the change in function for this mutant.

Cardiac α-myosin isolated from the hearts of homozygous transgenic mice engineered to carry the R403Q mutation showed enhanced average force, actin filament velocity and ATPase activity, consistent with a gain in myosin function [14]. These findings suggested a molecular mechanism for the supra normal cardiac performance that is often evident in humans with hypertrophic cardiomyopathy [2]. However, gain in function can have detrimental physiological effects. If the cardiac sarcomere is designed to function within a normal range of physiological force development, then higher levels of average force could cause the sarcomeric and myocyte disarray seen in FHC-diseased hearts [3], [16]. Could this characteristic FHC histology be initiated by structural changes in the actomyosin complex which disrupts myofibril assembly, without other effectors contributing?

It was shown that actively contracting cardiomyocytes expressing GFP-myosin (green fluorescent protein fused to an embryonic myosin heavy chain) carrying the R403Q mutation, exhibited a significant decrease in organization of the contractile cytoskeleton of embryonic chicken cardiomyocytes [36]. The FHC mutations studied (including the R403Q), all of which are known to affect myosin motor activity, disrupted the myofibril organization in a manner that is characteristic of this disease. Similarly, cardiomyocytes isolated from heterozygous α-myosin^R403Q/+^ trangenic mice showed far fewer well-aligned parallel myofibrils than the wild-type control [37]. These data are consistent with a direct effect of the R403Q myosin mutation on the organization of the contractile cytoskeleton.

Here we present structural evidence for the molecular mechanism leading to this morphological disarray. The possibility exists that the structural differences observed here may be isoform-specific; however, we favor the view that the morphological disarray characteristic of cardiac myosin R403Q-caused FHC, is a direct outcome of molecular level conformational variability in the actomyosin modes of interactions. Although there may be an improvement in motor performance of individual R403Q molecules, heterogeneous mixture of myosin heads with different cycling kinetics and large conformational variability within the sarcomere would likely uncouple the mechanical coordination that normally occurs between the myosin heads, a condition that may not only affect myofibril morphology, but may also alter ventricular performance and thereby contribute to a compensatory hypertrophy in cardiac muscle tissue.

## Materials and Methods

### Protein Preparations

Actin was prepared from chicken pectoralis acetone powder [38] and stored at 4 °C as F-actin (10–15 mg ml^−1^) in 5 mM KCl, 5 mM imidazole, pH 7.5, 2 mM MgCl_2_, 3 mM NaN_3_. It was generally used within 2–3 weeks of preparation. Just prior to application to the glow-discharged 400-mesh copper grids coated with holey carbon film, F-actin was diluted to 0.025–0.03 mg ml^−1^ with 20 mM NaCl, 5 mM NaP_i_, pH 7.0, 1 mM MgCl_2_, 1 mM EGTA, 2 mM NaN_3_.

Smooth muscle S1 containing the regulatory and essential light chains was expressed in an insect cell line as described [39]. Smooth muscle S1 containing the R406Q point mutation (the equivalent to the R403Q in cardiac myosin) was expressed under identical conditions to wild-type. A ‘Flag’ tag was ligated to cDNA encoding 852 amino acids for S1, to facilitate purification. The S1 was diluted to 0.5 mg ml^−1^ in 10 mM NaCl, 10 mM imidazole, pH 7.0, 1 mM MgCl_2_, 1 mM dithiothreitol (DTT) in the absence of nucleotide. S1 in the presence of 0.5 mM MgADP was used at the higher concentration of 2.0 mg ml^−1^ in the same buffer to compensate for the reduced binding affinity.

### Actomyosin Complexes

Thirty minutes prior to the preparations of the electron microscopy grids, the ADP myosin samples were incubated in 10 mM imidazole pH 7.0, 10 mM NaCl, 1 mM MgCl_2_, 1 mM DTT, 3 mM NaN_3_ and 0.5 mM ADP on ice. Then after 1 min incubation of filamentous actin in a humid chamber, the grids were rinsed twice with the respective myosin buffer without the myosin sample. The myosin sample, diluted to ∼0.5 or 2 mg/ml in their respective dilution buffer, were applied to copper grids coated with Quantifoil holey carbon films (Quantifoil Micro Tools GmbH, Jena, Germany) for 30 s, and replaced by an additional drop of sample (30 s). The excess of liquid was blotted, and the grids were plunged into liquid ethane cooled by liquid N_2_.

### Electron Microscopy

Low-dose images were recorded with a Tecnai G2 T12 electron microscope (FEI Electron Optics, Eindhoven, the Netherlands), using the 626DH cryo-holder (Gatan Inc, Pleasanton, CA) at a nominal magnification of 52,000×, accelerating voltage of 120 keV, at 1.5 µm defocus and with a total electron dose of 10 e^−^/Å^2^. Micrographs were digitized with a SCAI scanner (Integraph, Phoenix, AR) with a pixel size of 0.27 nm on the sample. Data were taken for wild-type as well as for the R403Q mutation using different biochemical preparations, in the absence and presence of MgADP. Optical diffraction and manual inspection were used to ascertain the quality of the images that were included in the data sets (i.e. well defined Thon rings, no drift and no astigmatism). In addition, the optical diffraction patterns of the selected filaments were used as filtering criteria for the quality of the data (well defined layer lines). To ensure that the observed differences are not due to sample preparation artifacts, the wild-type and mutant samples were prepared for data collection in identical ways by the same operator.

### Helical Reconstructions

The Brandeis Helical Imaging Package [40] provided the alignment parameters for each filament that was introduced in the real space average. These include phase origin and particle tilt. The parameters were refined through minimization of the phase error in reciprocal space [41]. Using these alignment parameters, 3D maps were computed separately for each individual filament in the data set [8], [42]. All reconstructions included 23 layer lines that were trimmed to 2.1 nm resolution. Since this is within the first node of the contrast transfer function, no phase correction was necessary. The abrupt edge in the data introduced by this procedure was smoothed to zero using a Gaussian fall off. The layer line orders used were 2, −11, 4, −9, 6, −7, 8, −5, −3, −1, 14, 1, −12, 3, 5, −8, 7, −6, −4, −2, 13, 0 and the equator. The data were divided into two clusters respectively, using the similarity of the layerline patterns as a clustering criterion. The individual filament maps were aligned in real space, normalized, and averaged [42], [43]. Wild-type reconstructions in the apo and the nucleotide states were indistinguishable from those we previously obtained [8].

### Reconstruction by Iterative Helical Real Space Refinement

The iterative helical real space refinement (IHRSR) method [28] is a hybrid approach that uses real-space, single-particle processing and imposition of helical symmetry in an iterative manner. Our implementation uses EMAN [44] for the single-particle reconstruction portion and routines adapted from the CoAn suite [6], [45] to determine and impose the helical symmetry. This implementation was extensively tested with calculated and experimental data from frozen-hydrated actin-myosin [see Supplemental Data in 7]. For this study, a box size of 80 × 80 pixels with a 0.54 nm pixel size was used. This corresponds to about 15 asymmetric units of the helix, a little over one actin crossover. An overlap of 60 pixels was chosen, allowing every asymmetric unit to contribute to four different views of the helix. Maps were calculated for all samples using the clustered sub-data from the helical reconstructions described above. The variance for the IHRSR maps (necessary for calculating t-tests) in each state was estimated using the differences between the two respective clusters. IHRSR maps were also calculated for previously obtained data of actin decorated with wild-type smooth muscle myosin in the presence of ADP and in the absence of nucleotide [8]. These reconstructions were indistinguishable from the IHRSR reconstructions obtained from the wild-type samples used for this work. Statistical significance of features in difference maps was assessed using a t-test procedure [46].

### Sorting of structural states

The IHRSR method allows for sorting structural states provided that some short-range order is present and the states are correlated over the size of the box (about one actin cross-over for this study). We built a variety of alternative models into the R403Q based densities and calculated density maps for sorting the experimental data. There was no significant difference between any of the reconstructions based on sorted data and that based on the complete data, and no significant difference between the sorted reconstructions.

### Structural flexibility mapping

The absolute value of individual differences (AVID) was used to map partial occupancy within the filaments [29]). This procedure was applied to all maps generated by the helical reconstruction technique. The final AVID maps were generated by averaging over the four AVID maps for each condition. Peaks further removed than 2 nm from features in the corresponding density maps (density larger than 0.5 standard deviations above mean) were removed.

### Density segmentation

Segmentation was performed using the 3D watershed transform [33]. This technique segments the density into self-consistent regions using the density information only, without prior knowledge of the underlying structure. The angle of the light-chain domain was determined by defining a line (skeleton) that has the same distance to the upper and lower boundary of the individual myosin segments or density boundaries.

### Modeling of the angular disorder

The angular disorder of the attachment angle was modeled using the atomic models obtained for wild-type smooth muscle actomyosin [8]. For this purpose, it was assumed that the hydrophobic anchor site identified at the helix-turn-helix motif of the lower 50kDa domain of myosin remains rigidly attached to the actin filament. Thus, the α-carbon coordinate of residue Pro^548^ was used as a pivot point to simulate angular disorder. Samples of size 30 were drawn from zero-mean Gaussian distributions with standard deviations of 5, 10, 15, 20, 25 and 30. The values within these samples were applied as rotations perpendicular to the filament axis to the atomic model of actin-bound myosin, using Pro^548^ as the pivot of the rotation, yielding 30 randomly rotated models for each standard deviation. Density maps were calculated from each model within these sets and an average of all 30 maps was generated for each standard deviation. These averages were compared with the experimental reconstructions of the R403Q samples. For both nucleotide states, the average of the set using a standard deviation of 15 gave the best match, indicating that the angular disorder is ∼15°.
